# Bushen Culuan Decoction Ameliorates Premature Ovarian Insufficiency by Acting on the Nrf2/ARE Signaling Pathway to Alleviate Oxidative Stress

**DOI:** 10.3389/fphar.2022.857932

**Published:** 2022-04-06

**Authors:** Yanxia Chen, Xiaodi Fan, Kun Ma, Kaili Wang, Caidie Tian, Min Li, Linjuan Gong

**Affiliations:** ^1^ Xiyuan Hospital, China Academy of Chinese Medical Science, Beijing, China; ^2^ Institute of Basic Medical Science of Xiyuan Hospital, China Academy of Chinese Medical Science, Beijing, China; ^3^ China Academy of Chinese Medical Science, Beijing, China

**Keywords:** Bushen Culuan Decoction, premature ovarian insufficiency, oxidative stress, Nrf2/ARE, Tripterygium wilfordii polyglycosidium

## Abstract

Premature ovarian insufficiency (POI) can result in lower fertility and shorten the female reproductive span. Bushen-Culuan Decoction (BCD) is a traditional Chinese medication utilized for treating POI for many years. We previously observed that BCD protects against further deterioration of the ovarian reserve of POI patients, however, the underlying mechanism has not been well studied. Our investigation seeks to evaluate the effect of BCD on POI induced by Tripterygium wilfordii polyglycosidium (TWP) and the likely mechanistic pathways, which we hypothesize may involve the Nrf2/ARE pathway. The body weights, estrous cycle, serum hormone levels, histological follicular analysis and quantification, levels of oxidative stress biomarkers in the ovarian tissue of POI mice models were evaluated. Western blotting and RT-PCR enabled quantification of the components of the Nrf2/ARE pathway. Our results showed that BCD restored hormonal profiles and estrous cycles of POI mice similar to those observed in healthy controls. BCD reduced the numbers of atretic follicles while increasing the number of primordial follicles. BCD facilitated lower 8-OHdG and MDA levels while increasing levels of key antioxidant enzymes including GSH-Px, CAT, and SOD. Furthermore, TWP increased Bach 1, Nrf2, and Keap 1 expressions at the translational level, while decreased that of HO-1. BCD treatment also promoted nuclear translocation rates of Bach 1 and Nrf2, suppressed Keap 1 protein expression, as well as raised HO-1 protein expression. Taken together, BCD likely augments ovarian reserve by activating the Nrf2/ARE signaling pathway, which stimulated higher levels of antioxidants and suppressed oxidative stress. BCD may be an important therapeutic compound in POI.

## Introduction

As an increasing proportion of women in modern society postpone childbearing due to lifestyle, career and financial constraints, premature ovarian insufficiency (POI) has become an increasingly common clinical occurrence. POI mainly occurs in women younger than 40 years old who experience early depletion of ovarian follicular reserve (Liu et al., 2019). POI is characterized by primary or secondary menstrual disturbance with hypergonadotropism and hypoestrogenism (Domniz and Meriow, 2019). The prevalence of spontaneous POI is approximately 1% (Tsiligiannis et al., 2019), and exerts devastating effects on the physical and psychological well-being of affected women. Without any intervention, it will develop into premature ovarian failure rapidly, and cause many long-term complications, including vasomotor symptoms, urogenital atrophy, osteoporosis and fracture, cardiovascular disease, and increased all-cause mortality. However, the cause of POI is obscure in a significant proportion of cases and may be related to factors such as genetic abnormalities, toxicities, environmental factors, autoimmune, and iatrogenic causes (Sharif et al., 2019).

Tripterygium wilfordii polyglycosidium (TWP) is well known for its role in treating inflammatory and autoimmune conditions. However, this component is notorious for inflicting severe ovarian toxicity, resulting in premature menopause and sterility (Guo et al., 2019). Previous studies have suggested that TWP can directly destroy ovarian follicles leading to a depleted primordial follicle pool (Xu et al., 2017; Yuan et al., 2019), consequently predisposing women to POI (Ai et al., 2018; Yang et al., 2019). Previous research suggests that TWP could cause target organ toxicity via induction of metabolic dysfunction, mitochondrial disruption, apoptosis, autophagy, and oxidative stress. Of these, oxidative stress appears to be a key molecular mechanism (Xi et al., 2017; Pan W. et al., 2019). Other studies further highlight the significant role of oxidative stress in TWP-induced POI (Ma et al., 2014; Fang et al., 2019).

Oxidative stress arises when reactive oxygen species (ROS) overwhelms the protective capacity of intrinsic antioxidant mechanisms, resulting in DNA, protein, and fat destruction of cell. Byproducts of oxidative stress damage include 8-hydroxy-deoxyguanosine (8-OHdG), which represents oxidized DNA, and malondialdehyde (MDA), a lipid peroxidative byproduct, all of which impairs cellular homeostasis (Aitken, 2019). The nuclear factor-erythroid 2-related factor 2 (Nrf2) represents a basic leucine zipper (b ZIP) transcription factor and is responsible for regulating expression of many important antioxidant enzymes, thus providing vital cellular protection against oxidative stress (Li et al., 2018). Kelch-like ECH-associated protein 1 (Keap1), a molecule that negatively regulates Nrf2, contains six domains which comprises the Kelch domain and broad complex-tramtrack-bric-a-brac (BTB) domain, of which the Kelch domain is responsible for interacting with Nrf2, and the BTB domain is involved in recruiting Cullin3 (Cul3) (Tu et al., 2019). BTB and CNC homology 1 (Bach 1) represents a heme-binding transcription factor that is able to competitively bind to small musculoaponeurotic fibrosarcoma oncogene homolog (Maf) family members with Nrf2 to form a heterodimer, leading to downregulated expression of antioxidant enzyme genes (Ochiai et al., 2008).

In physiological conditions, Nrf2 is primarily localized in the cytoplasm with a relatively low nuclear level. This is because Nrf2 is incorporated into the Keap1-Cul3 complex in the cytoplasm, resulting in increased proteasomal degradation and ubiquitination (Akino et al., 2018; Lu et al., 2018). Conversely, Bach 1 is mainly distributed in the nucleus and combines with Maf elements to inhibit natural antioxidants by modulating the antioxidant response element (ARE) (Yamaoka et al., 2017). Oxidative stress releases Nrf2 from the Nrf2-Keap1-Cul3 complex, allowing Nrf2 to accumulate and translocate into the nucleus to form the Nrf2-Maf-ARE complex that will induce gene expression of protective antioxidant enzymes, which includes glutathione (GSH), heme oxygenase-1 (HO-1), superoxide dismutase (SOD), and catalase (CAT) (Li et al., 2018; Xu et al., 2018; Akino et al., 2019).

POI therapy and prevention may be alleviated by traditional Chinese medicines (TCM). TCM has a long history of being used to treat POI in China. Based on TCM theory, POI is believed to originate in the Chong and Ren Meridians, with key pathogenic factors being Ben-Xu (kidney insufficiency) and Biao-Shi (blood stasis). Therefore, the principle of TCM treatment for POI is lies in activating blood circulation as well as tonifying the kidney. The Bushen-Culuan Decoction (BCD) is made from a combination of 12 herbs: Gou Qi Zi (Lycii fructus), Tu Si Zi (Cuscutae Semen), Sang Ji Sheng (Talxilli Herba), Nv Zhen Zi (Ligustri Lucidi Fructus), Chuan Niu Xi (Cyathulae Radix), Dang Gui (Angelica sinensis), Xu Duan (Dipsaci Radix), Chi Shao (Paeoniae Radix Rubra), Ze Lan (Lycopi Herba), Pu Huang (Typhae Polien), Dan Shen (Salviae Miltiorrhizae Radix ET Rhizoma), and Xiang Fu (Cyperi Rhizoma). Previous clinical trials have been showed that BCD can alleviate the clinical symptoms of POI, improve gonadal hormone profiles while elevating antral follicular counts in POI patients (Ma et al., 2015; Ma et al., 2019). Given the wealth of information, the molecular workings of BCD in the treatment of POI are complicated and multifaceted and is thus not fully understood. The current study examines how BCD affects the Nrf2/ARE signaling pathway in order to delineate the mechanisms underlying the therapeutic effects of BCD in POI.

## Methods and Animals

### Animals

Female Balb/c mice (*n* = 100, weighing 18–20 g each) and 6-week-old CD-1 mice (*n* = 40, half male and female) were obtained from Beijing Vital River Laboratory Animal Technology Co., Ltd. (Certificate number: SCXK (Beijing) 2017-0033). Animals were reared in specific-pathogen-free facilities under controlled light-dark exposure (12-h each of dark and light), temperatures (20–24°C), and humidity (45%–70%). Ad libitum food and water were provided. Animals were handled strictly adhering to the Guidelines for the Care and Use of Laboratory Animals. Ethical approval was obtained from the Ethics Committee of Xi Yuan Hospital of China Academy of Chinese Medical Sciences.

### Preparation of Bushen-Culuan Decoction Formula Granules

The herbs of BCD were purchased from Xiyuan Hospital of China Academy of Chinese Medical Science [supplied by Hebei Baicaokang Pharmaeutical Co., Ltd. (Heibei, China, Drug GMP certificate: HE20150001, Drug Manufacturing Certificate: HE20190064)]. BCD component herbs were all verified and conformed to the standard of quality in the 2015 Chinese Pharmacopoeia. Herbs were authenticated based on the following parameters: thin-layer chromatography, chemical, microscopic, and morphological identification, as well as detection of heavy metal and deleterious elements. A pharmaceutical company [Beijing Tcmages Pharmaceutical Co., Ltd. (Beijing, China, Drug GMP certificate: BJ20180373, Drug Manufacturing Certificate: BJ20180032)] was enlisted to produce BCD granules made out of a concentrated mixture of the components presented in Table 1.

**TABLE 1:** Information of components in Bushen Culuan Decoction.

Chinese name	Botanical name	Common name	Family	Part used	Weight/g
Tusizi	*Cuscuta chinensis* Lam.	Cuscutae Semen	Convolvulaceae	Ripe seed	15
Nvzhenzi	*Ligustrum lucidum* W.T.Aiton	Ligustri Lucidi Fructus	Oleaceae	Ripe fruit	15
Gouqizi	*Lycium chinense* Mill.	Lycii Fructus	Solanaceae	Ripe fruit	15
Sangjisheng	*Taxillus chinensis* (DC.) Danser	Talxilli Herba	Loranthaceae	Branch, with leaf	15
Xuduan	*Dipsacus asper* Wall. ex DC.	Dipsaci Radix	Caprifoliaceae	Root	15
Chuanniuxi	*Cyathula officinalis* K.C.Kuan	Cyathulae Radix	Amaranthaceae	Root	15
Danggui	*Angelica sinensi* (Oliv.) Diels	Angelicae Sinensis Radix	Apiaceae	Root	10
Chishao	*Paeonia lactiflora* Pall.	Paeoniae Radix Alba	Paeoniaceae	Root	15
Zelan	Lycopus lucidus var. hirtus (Regel) Makino & Nemoto	Lycopi Herba	Lamiaceae	Aerial part	15
Puhuang	*Typha angustifolia* L.	Typhae Pollen	Typhaceae	Pollen	10
Danshen	*Salvia miltiorrhiza* Bunge	Salviae Miltiorrhizae Radix et Rhizoma	Lamiaceae	Root and rhizome	15
Xiangfu	*Cyperus rotundus* L.	Cyperi Rhizoma	Cyperaceae	Rhizome	10

### High-Performance Liquid Chromatography Analysis of Bushen-Culuan Decoction

BCD granule stability and quality were evaluated utilizing a high-performance liquid chromatography (HPLC) system (Agilent 1100, USA) equipped with a Waters Xbridge-C18 analytical column (4.6 × 150 mm, 5 μm). Mobile phase A indicated the acetonitrile organic phase, while mobile phase B indicated the ultrapure water (containing 0.1% phosphoric acid) aqueous phase. Mobile phases were altered based on the following: 0–5 min, 10% (phase A); 5–35 min, 10%–20% (phase A); 35–50 min, 20%–30% (phase A); 50–55 min, 30%–37% (phase A), 55–60 min, 37%–10% (phase A). The flow rate was 1.0 ml/min and the UV spectrum was calibrated to 230 nm. Specnuezhenide (CAS: 39011-92-2), cyasterone (CAS: 17086-76-9), ferulic acid (CAS: 537-98-4) and isorhamnetin-3-O-neohesperidoside (CAS: 55033-90-4) were procured from National Institutes for Food and Drug Control (Beijing, China), and hyperoside (CAS: 482-36-0), asperosaponin VI (CAS: 39524-08-8), peoniflorin (CAS: 23180-57-6) and typhaneoside (CAS: 104472-68-6) were obtained from Shanghai Standard Technology Co., Ltd. (Shanghai, China). The detection wavelength of BCD was identified based on retention time compared with reference substance.

### Establishing a Premature Ovarian Insufficiency Mouse Model and the Treatment

The chemical constituents of TWP tablets (Grand Pharmaceutical Huangshi Feiyun Pharmaceutical Co., Ltd., Lot NO. Z42021212) were normalized and evaluated as Qu L.’s research (Qu et al., 2015). TWP, BCD, medroxyprogesterone acetate (Zhejiang Xianju Pharmaceutical Co., Ltd., Lot NO. H33020715), and estradiol valerate (Bayer Healthcare Co., Ltd., Lot NO. J20171038) were dissolved in distilled water containing 0.25% Tween 80 and 0.2% carboxymethyl cellulose sodium. Final concentration of TWP was 4 mg/ml, BCD was 0.27 g/ml, estradiol valerate was 0.015 mg/ml, and medroxyprogesterone was 0.09 mg/ml respectively. A 4°C refrigerator was used to store these compounds until further used.

**FIGURE 1:**
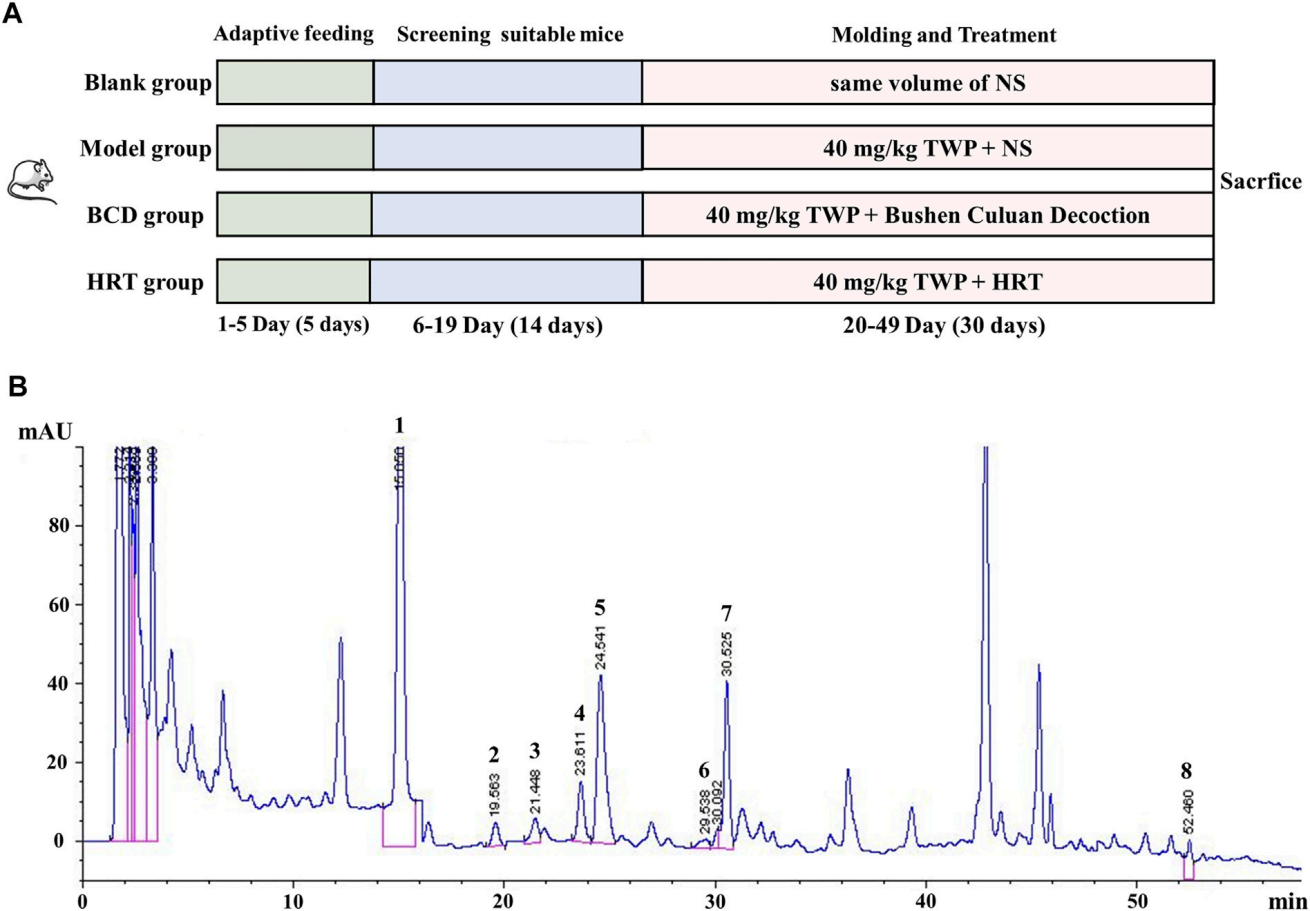
The experimental schema and representative HPLC chromatogram of BCD. **(A)** POI mouse model development and treatment protocol. **(B)** HPLC analysis of BCD: Mobile phase was acetonitrile/ultrapure water (containing 0.1% phosphoric acid) gradient for 60 min with a flow of 1 ml/min. Peaks: 1. peoniflorin, 2. ferulic acid, 3. typhaneoside, 4. hyperoside, 5. isorhamnetin-3-O-neohesperidoside, 6. specnuezhenide, 7. cyasterone and 8. asperosaponin VI.

Mice were allowed 5 days of adaptive diets and has their estrous cycles continuously observed for 14 days. Eighty female mice noted to have relatively regular estrous cycles (5–6 days) were selected, with 20 mice each designated in the hormone replacement therapy (HRT), BCD, Model, and Blank groups. In accordance with previous literature and our initial modeling experiments, mice in the HRT, BCD, and Model groups were intragastrically fed 40 mg/kg/d TWP suspension every morning at 8 a.m. for 30 days to induce POI (Lu et al., 2014; Huang et al., 2018; Yuan et al., 2019). The Blank group underwent the same procedure but with normal saline (NS) given instead. Before this experiment, we carried out the concentration of BCD screening research. Based on these findings, an oral BCD suspension at a dose of 2.67 g/kg/d was administered to the mice in the BCD group at 4 p.m. every afternoon during developing the POI mouse model. At the same time, the mice in the HRT group were treated with 4 days of oral 0.15 mg/kg/d estradiol valerate suspension, followed by the addition of 0.91 mg/kg/d medroxyprogesterone acetate suspension daily on the fourth day onwards, with one rest day from hormonal treatments every 4 days. The Blank and Model groups underwent the same procedure but received the same volume of NS instead (Figure 1A).

### Animal Samples Collection

At the end of the experimental period, all mice models were starved overnight before being weighed and anesthetized the following morning with intraperitoneal 1% pentobarbital sodium (50 mg/kg) injections. Prior to euthanizing the animals, we extracted serum samples for further hormonal ELISA tests. The thymus, lung, cardiac, liver, spleen, kidney, uterus and ovary were then dissected to determine the organ index (organ index = organ weight/body weight × 100%). One ovary from each mouse was frozen in liquid nitrogen and stored at −80°C for further analysis while the other ovary was fixed with 4% paraformaldehyde (PFA) for histology and immunostaining.

### Estrous Cycle Monitoring

Ten mice chosen at random from every group had their estrous cycle checked daily and body weights monitored every 4 days during the experimental period. Estrous cycle staging was done by vaginal smear cytological examination every morning between 9 a.m. and 10 a.m. Smears were obtained by flushing 20 µl of sterile NS into the vaginal cavity, spreading the flushed fluid on a glass slide, and fixing the samples with 95% ethanol for 30 min. The Wright-Giemsa Stain solution (Solarbio Science & Technology Co., Ltd., Beijing, China) was used to stain the slides, which were then rinsed with running water, air-dried, and then observed using a light microscope at ×100 magnification.

### Serum Sexual Hormone Test

Hormonal assays to determine the levels of serum anti-mullerian hormone (AMH, CUSABIO, CSB-E13156), inhibin-B (INH-B, CUSABIO, CSB-E08151), estradiol (E_2_, CUSABIO, CSB-E05109), luteinizing hormone (LH, CUSABIO, CSB-E12770), and follicle stimulating hormone (FSH, CUSABIO, CSB-E06871) were evaluated using mouse-specific ELISA kits strictly following instructions provided by the manufacturer. The sensitivity of these kits was defined as the lowest mouse concentration that could be differentiated from zero and the CV% was less than 15%. Results were based on the 450 nm absorbance.

### Histological Examinations

Histopathological examination was carried out on ovarian and uterine samples fixed in 4% PFA, dehydrated with ethanol and cleansed with xylene prior to being used to produce 5 μm slices. Samples were rehydrated before being treated with hematoxylin and eosin (HE) for further morphological observation and quantification of ovarian follicles. Two independent investigators quantified follicles by counting the number of follicles on whole ovarian three serial sections. Primordial follicles were visualized using ×400 magnification while all primary, secondary and antral follicles were observed at ×200 magnification.

### Biochemical Estimations of Oxidative Stress Markers

Ovarian tissue was homogenized in PBS to produce 10% ovarian homogenate, before being centrifuged for 10 min at 4°C and 3,000 rpm to collect the supernatant. 8-OHdG (CUSABIO, CSB-E10527), MDA (Njjcbio, A003-1-2) and CAT (CUSABIO, CSB-E14190) levels, and the activities of GSH-Px (Njjcbio, A005-1-3) and SOD (CUSABIO, CSB-E08556) in the ovarian homogenate supernatant were performed in accordance with protocols provided by the manufacturer.

### Real-Time qPCR Analysis

Ovarian expression of the Bach 1, Nrf2, Keap 1, and HO-1 genes were evaluated using real-time qPCR. Fifty milligrams of ovarian tissues with TRIzol reagent (Invitrogen) on ice before RNA extraction that was carried out based on instructions provided by the manufacturer. Eight microliter RNA was used for 1% agarose gel electrophoresis. Twenty microliter of TIANScript reverse transcriptase (RT) mix, sequence-specific PCR primers, and RNA were combined for analysis using the Prism 7500 sequence detection system. Final results were evaluated using the ABI Prism 7500 SDS software (ABI7500, USA) and a SYBR FAST qPCR Kit Master Mix (×2) Universal kit (Biosystems). The NCBI-Primer was used to design all primer sequences (shown in Table 2). The 2^−∆∆Ct^ method was utilized in calculating relative gene expression levels against endogenous control gene beta actin.

**TABLE 2:** The quantitative real-time RT-PCR primers.

Gene	ID number	Updated Date	Forward and Reverse Primer	Primer sequence	Amplified length (bp)
*Bach 1*	12013	14 January 2020	F	TGT​GCA​TAG​CAC​CAA​CGT​CT	155
			R	GGC​CTA​CGA​TTC​TCG​AGT​GG	
*Nrf2*	18024	9 February 2020	F	TGA​AGC​TCA​GCT​CGC​ATT​GA	108
			R	TGC​TCC​AGC​TCG​ACA​ATG​TT	
*Keap1*	50868	5 November 2019	F	GCC​CCG​GGA​CTC​TTA​TTG​TG	101
			R	TTAGGGGCCCCGCCAT	
*HO-1*	15368	9 February 2020	F	AAG​CTT​TTG​GGG​TCC​CTA​GC	98
			R	GGC​TGG​ATG​TGC​TTT​TGG​TG	
			R	CTT​GGA​TCC​AGA​CAA​GCA​GC	
*beta Actin*	11461	28 January 2020	F	TGA​GCT​GCG​TTT​TAC​ACC​CT	231
			R	TTT​GGG​GGA​TGT​TTG​CTC​CA	
			R	CAA​CCA​GAG​CAG​CAC​ACT​CTA	

### Western Blotting Analysis

Proteins were extracted from the four different mice groups. The cytoplasm and nucleus proteins were isolated using a Nucleus-cytoplasmic protein preparation kit (APPLYGEN, P1200). The BCA Protein Assay Kit (CoWin Biosciences) was used to quantify ovarian lysate protein concentrations. Proteins were separated via electrophoresis and immunoblotted onto polyvinylidenefluoride (PVDF) membranes. A 5% bovine serum albumin solution (BSA) was added to the membranes for an hour before being incubated at 4°C overnight with the following primary antibodies: Bach 1(Abcam, ab49657), Nrf2 (Abcam, ab62352), Keap 1 (Abcam, ab119403), HO-1 (Abcam, ab13248), β-actin (ZS, TA-09) and Histon H3 (all antibodies were at a dilution of 1:1,000 in blocking buffer). The next morning, we added horseradish peroxides (HRP)-conjugated secondary antibody to incubate at room temperature for another 40 min. Signals were detected by enhanced chemiluminescence and imaged with a Gel Image system ver.4.0 (Tanon, China). Protein levels were normalized first before measurement of the bands using the ImageJ 6.0 software program.

### Acute Toxicity Study of Bushen-Culuan Decoction Formula Granules

Forty 6-week-old CD-1 mice (half male and female) each weighing more than 18 g, were purchased from Beijing Vital River Laboratory Animal Technology Co., Ltd. (Certificate number: SCXK (Beijing) 2017-0033). All the mice were divided equally (*n* = 20 each) into BCD and control groups randomly after 3 days of adjustable feeding. The night before gavage, mice were fasted and only drank water. The BCD group mice were fed with the maximal concentration and maximal volume of BCD solution daily by intragastric administration, while control group mice underwent the same procedure but with NS instead. All mice were closely observed for 2 weeks for any potential adverse effects. Mice were weighed daily and their average daily feed intakes (ADFI) were recorded. After the 2-week observation period, mice were fasted overnight before they were anesthetized for further evaluation of their internal organs.

### Statistical Analysis

The SPSS 16.0 was used to perform all statistical analysis. Results were depicted in terms of mean ± SEM. One-way ANOVA or Fisher’s exact tests were used to analyze statistical differences between groups. *p* values of <0.05 or <0.01 were indicative of statistical significance.

## Results

### The High-Performance Liquid Chromatography Map of Bushen-Culuan Decoction

HPLC-fingerprint was conducted to identify the main ingredients of BCD granules. Eight major compounds were identified and determined in BCD granules, including peoniflorin (RT, 15.050 min), ferulic acid (RT, 19.563 min), typhaneoside (RT, 21.448 min), hyperoside (RT, 23.611 min), isorhamnetin-3-O-neohesperidoside (RT, 24.541 min), specnuezhenide (RT, 29.538 min), cyasterone (RT, 30.525 min), and asperosaponin VI (RT, 52.460 min). The representative chromatography was depicted in Figure 1B.

### General Mice Condition and Change in Mice Body Weights

**FIGURE 2:**
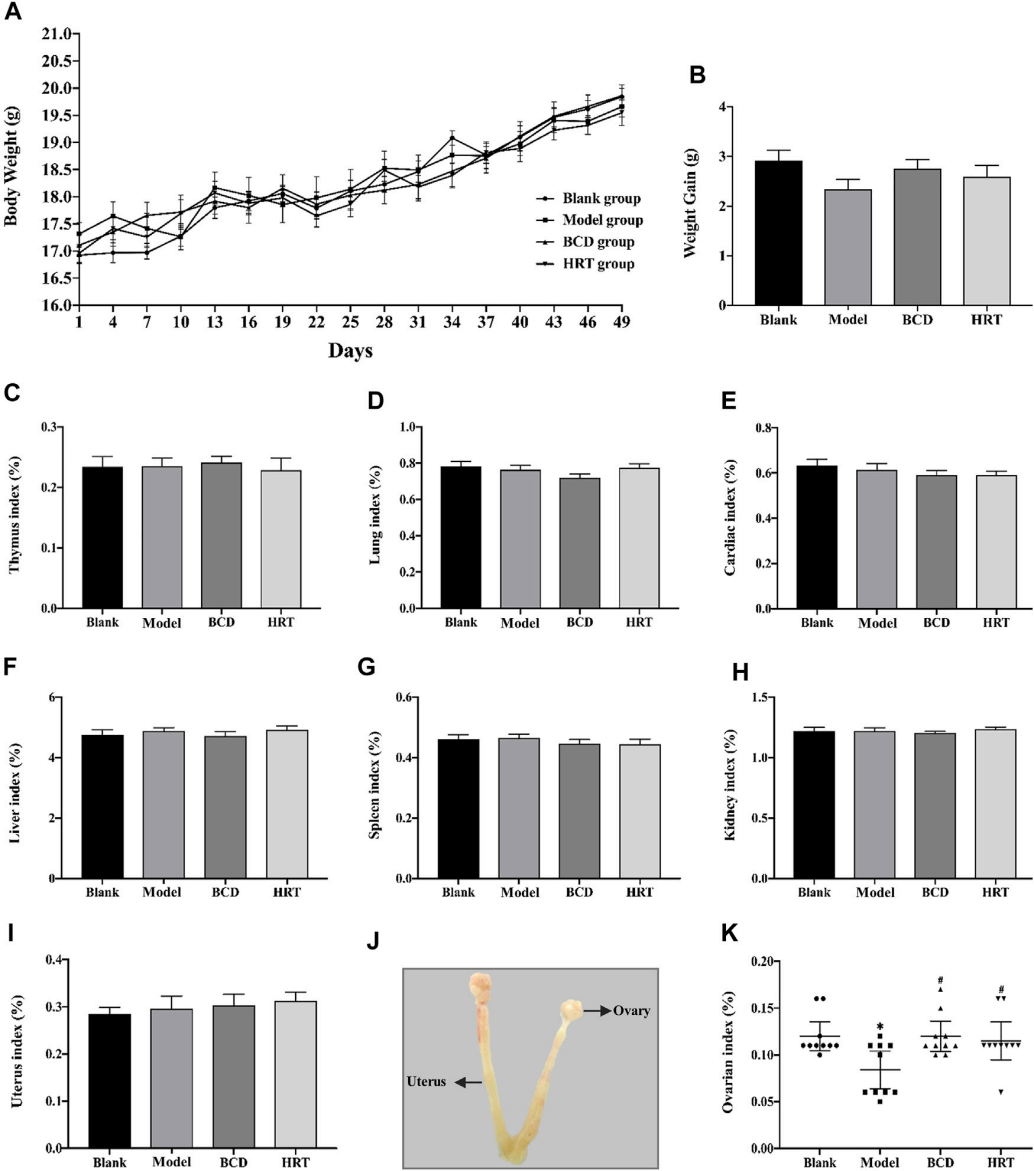
The impact of BCD on the body weight and organ indices of POI mice. **(A)** Body weights of mice of all four cohorts. Cumulative mice body weights in those of the Model, BCD, and HRT groups were all down-trending with TWP treatment during the first week. After this, all mice demonstrated increased body weights that were similar between all four groups. **(B)** Weight gain from baseline to sacrifice. **(C)** Thymus index. **(D)** Lung index. **(E)** Cardiac index. **(F)** Liver index. **(G)** Spleen index. **(H)** Kidney index. **(I)** Uterus index. **(J)** Uterus and ovarian anatomical diagram. **(K)** Ovarian indices of mice from all four groups. Data is visualized in terms of mean ± SEM. (*n* = 10, *p < 0.05, **p < 0.01, compared with the Blank group; ^#^ *p* < 0.05, ^##^ *p* < 0.01, compared with the Model group).

Before TWP treatment, all mice across the four groups had approximately equal weights. Total mice body weights after TWP treatment demonstrated a downward trend in the Model, BCD, and HRT groups. However, mice weights eventually increased progressively and no significant differences were seen in all four groups (Figure 2A). Weight gain did not differ significantly across all four groups at the end of the experiment (Figure 2B). Ratios of the thymus, lung, cardiac, liver, spleen, kidney, and uterus to body weights also did not differ notably (Figures 2C–J). One exception was the decreased ovarian index in the model group in comparison to the Blank groups (*p* < 0.05). Conversely, the ovarian index was elevated in the BCD and HRT groups (*p* < 0.05). The ovarian indices of the BCD and HRT groups were not significantly different (Figure 2K).

### Bushen-Culuan Decoction Improves Estrous Cycling in Premature Ovarian Insufficiency Mice

**FIGURE 3:**
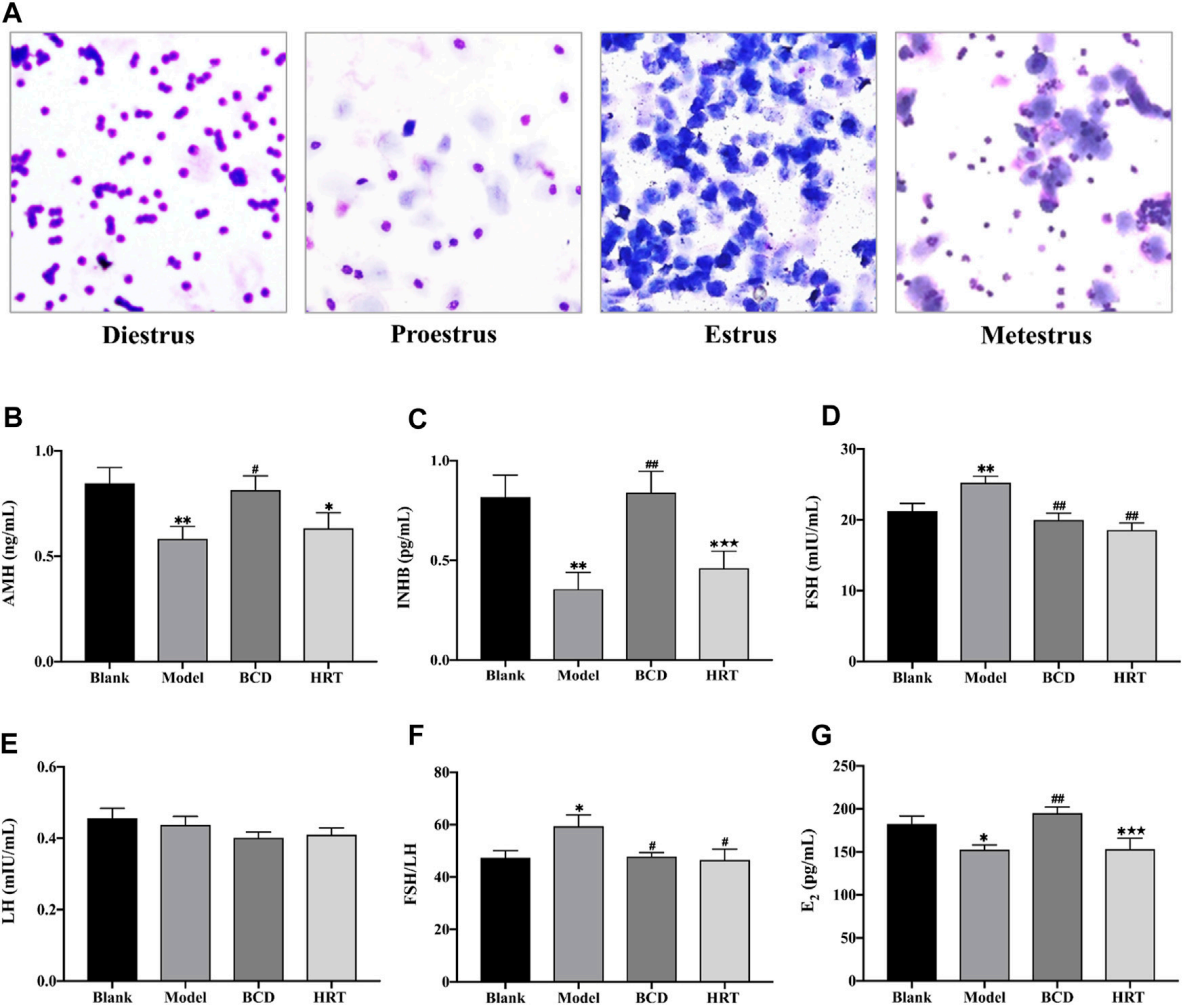
BCD normalizes estrous cycling and serum sex hormones. **(A)** Representative images of the estrous cycle phases. Briefly, diestrus is suggested with a significant proportion of leukocytes, proestrus by primarily nucleated epithelial cells, estrus by cornified epithelial cells, metestrus by leukocytes, nucleated epithelial cells, and cornified epithelial cells. BCD augments serum sex hormone levels in TWP-enhanced POI mice, and **(B)** AMH, **(C)** INHB, **(D)** FSH, **(E)** LH, **(F)** The ratio of FSH to LH and **(G)** E_2_ serum concentration were evaluated using ELISA. Data is visualized in means ± SEM. (*n* = 8, *p < 0.05, **p < 0.01, compared with the Blank group; ^#^ *p* < 0.05, ^##^ *p* < 0.01, compared with the Model group; ★*p* < 0.05, ★★*p* < 0.01, compared with the BCD group).

Estrous cycle stages were determined based on cell type, number, and morphology (Figure 3A). The estrous cycles of all mice were similar prior to TWP treatment. TWP-exposed mice demonstrated increased estrous cycle disturbances in contrast to mice of the Blank group. BCD treatment restored normal estrous cycles in half of the POI mice models in the BCD group. Estrogen secretion plateaued in the HRT group while those in the Model group continuously displayed abnormal hormonal profiles.

### Bushen-Culuan Decoction Can Restore Normal Serum Sex Hormone Levels in Premature Ovarian Insufficiency Mice

Serum AMH, INHB, and E2 levels of Model group mice were suppressed after TWP treatment (*p* < 0.01, *p* < 0.01, *p* < 0.05), however, these same mice possessed higher FSH level and ratio of FSH to LH in comparison to the Blank group (*p* < 0.01, *p* < 0.05). BCD intervention resulted in raised AMH, INHB, and E2 levels in comparison to the Model group (*p* < 0.05, *p* < 0.01, *p* < 0.01). BCD intervention also resulted in lower FSH level and FSH to LH ratio (*p* < 0.01, *p* < 0.05). Conversely, mice in the HRT group displayed increasing levels of AMH and INHB, but decreased FSH level and FSH to LH ratio (*p* < 0.01, *p* < 0.05). The differences between INHB and E2 levels of the BCD and HRT groups achieved statistical significance (*p* < 0.01) (Figures 3B–G).

### Bushen-Culuan Decoction Improves Ovarian and Uterine Histology of Premature Ovarian Insufficiency Mice

**FIGURE 4:**
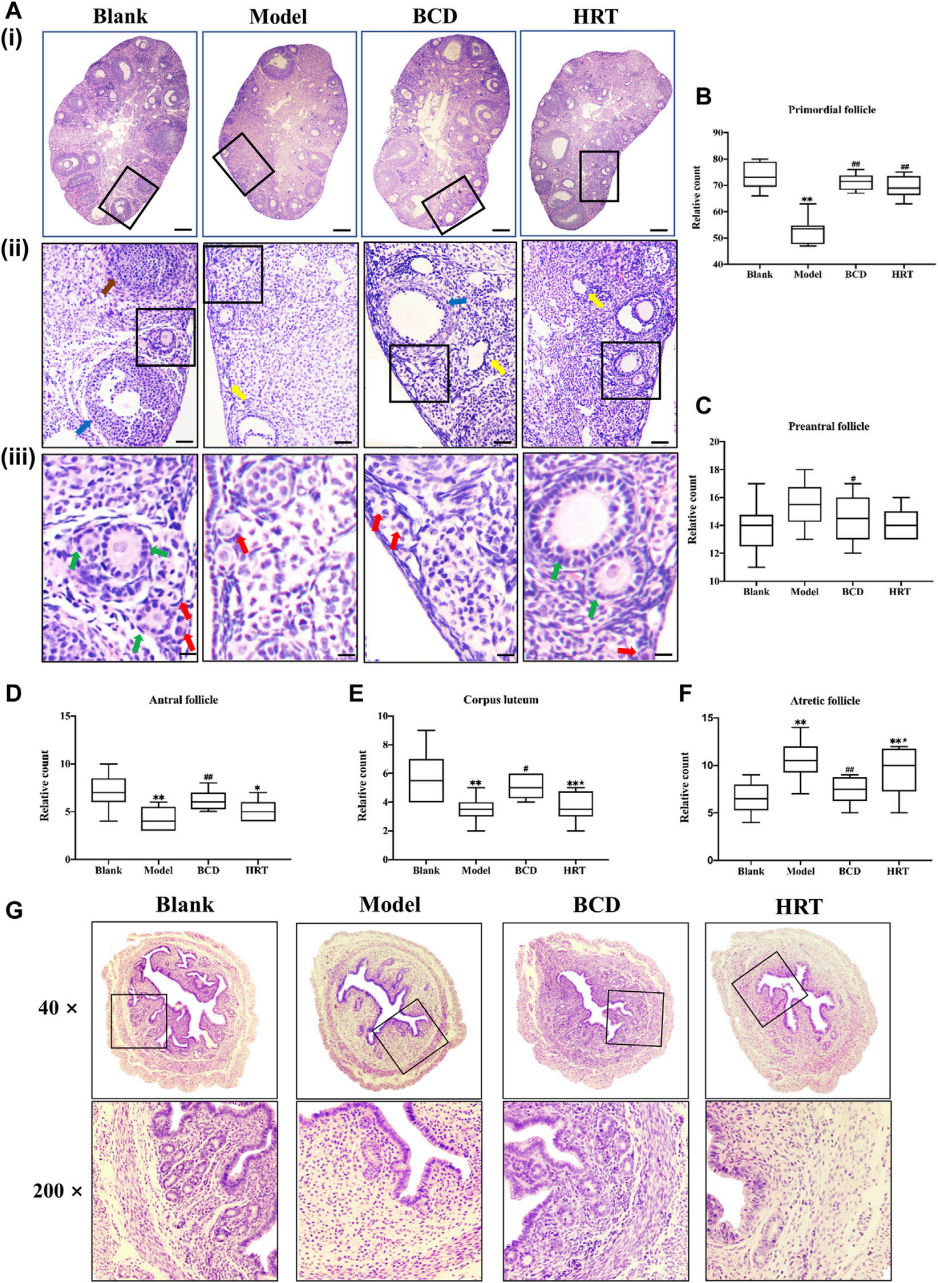
BCD protects the ovarian reserve and uterus of TWP-induced POI mice. **(A)** HE-stained ovarian sections and their representative images are depicted (×40, ×100×, and ×400 magnification). The red arrow shows primordial follicles, the green arrow shows preantral follicles, the blue arrow shows antral follicles, the yellow arrow shows atretic follicles, and the brown arrow shows a corpus luteum. The scale bars represent 200 μm **(i)**, 50 μm **(ii)** and 25 μm **(iii)**. Image analyses included three serial sections per mouse, which were then evaluated for the total primordial **(B)**, preantral **(C)**, antral **(D)**, corpus luteum **(E)**, and atretic **(F)**. **(G)** HE-stained uterine sections and their representative images are depicted (×40, ×200 magnification). Data is visualized in terms of means ± SEM. (*n* = 8, *p < 0.05, **p < 0.01, compared with the Blank group; ^#^ *p* < 0.05, ^##^ *p* < 0.01, compared with the Model group; ★*p* < 0.05, ★★*p* < 0.01, compared with the BCD group).

Figure 4A depicts histological analysis of the ovaries. TWP treatment effectively depleted numbers of dormant primordial follicles in contrast to the Blank group (*p* < 0.01), while HRT and BCD exposure restored them (*p* < 0.01) (Figure 4B). Preantral follicle numbers were significantly different between Model and BCD groups (Figure 4C). The Model group, however, had higher atretic follicles, in addition to lower antral follicles and corpus luteum in comparison to other groups (*p* < 0.01). Mice exposed to BCD had lower atretic follicles (*p* < 0.01), with higher antral follicle and corpus luteum numbers in contrast to the Model group (*p* < 0.01, *p* < 0.05). We also observed variabilities in corpus luteum and atretic follicles between the HRT and BCD groups (*p* < 0.05) (Figures 4D–F).

Uterine sections from the Blank group also had normal histopathological structures, specifically the adventitia, tunica muscularis, and mucous layer which made up almost two-thirds of uterine wall. There were also large numbers of normal glands. However, after TWP treatment, uterine sections from the Model group showed endometrium obvious thinning with decreased numbers of glandular organs. In comparison with the Model group, the endometrium was significantly thickened whilst a large number of glands were visible in BCD group. The histopathological structure in HRT group was also improved compared to Model group (Figure 4G).

### Bushen-Culuan Decoction Mitigates the Oxidative Damage in Ovary of Premature Ovarian Insufficiency Mice

**FIGURE 5:**
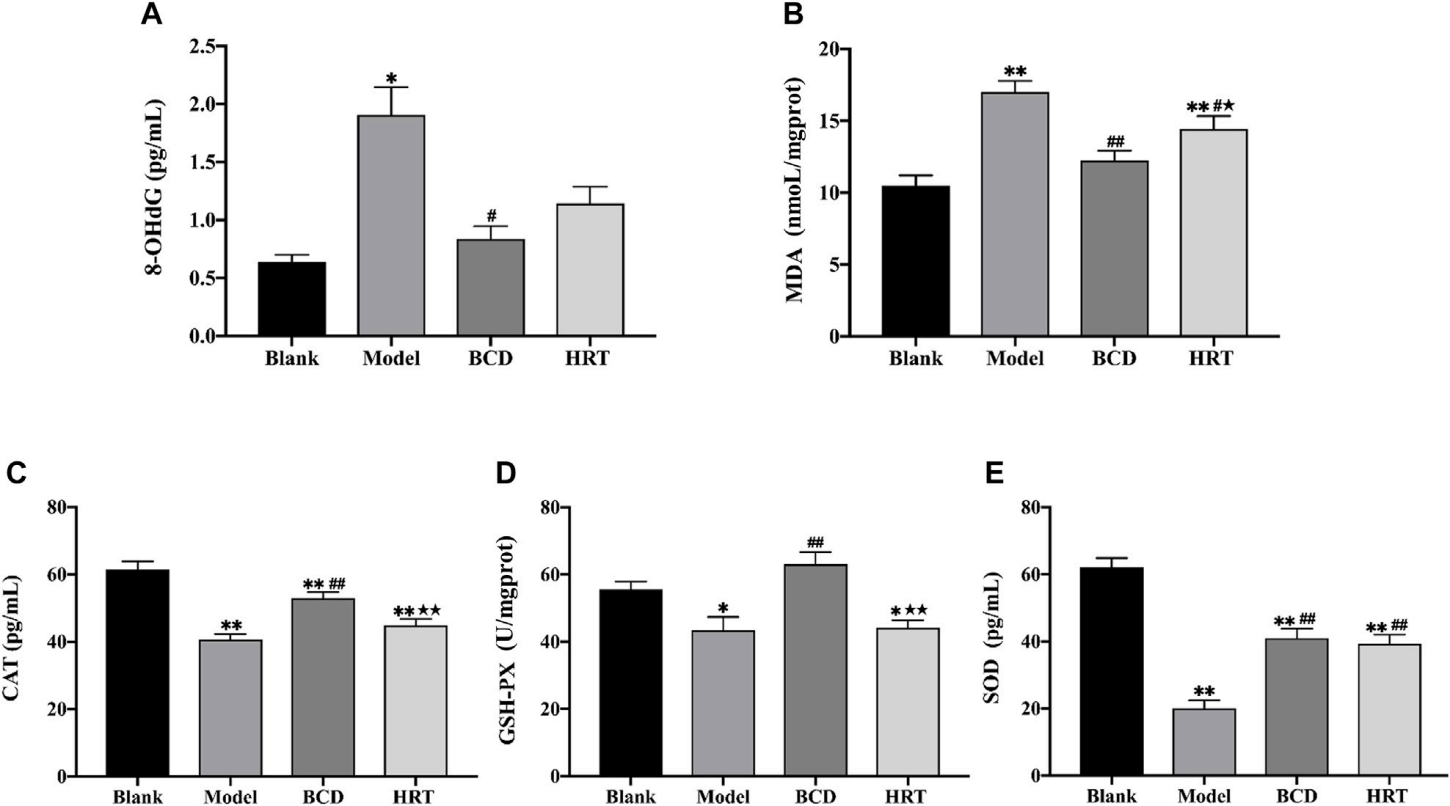
BCD alleviates oxidative stress in ovaries of POI mice. The relative contents or activities of **(A)** 8-OHdG, **(B)** MDA, **(C)** CAT, **(D)** GSH-PX and **(E)** SOD in ovarian homogenate supernatant were analysed. The data is visualized in terms of means ± SEM. (*p < 0.05, **p < 0.01, compared with the Blank group; ^#^ *p* < 0.05, ^##^ *p* < 0.01, compared with the Model group; ★*p* < 0.05, ★★*p* < 0.01, compared with the BCD group).

To evaluate the impact BCD in TWP-stimulated oxidative stress, the levels of 8-OHdG, MDA, CAT, GSH-Px, and SOD in ovarian tissues were measured. After TWP treatment, Model group mice demonstrated increased 8-OHdG and MDA levels (*p* < 0.05, *p* < 0.01), but lower levels of CAT, GSH-Px and SOD compared to the Blank group (*p* < 0.01, *p* < 0.05, *p* < 0.01). BCD intervention resulted in decreased 8-OHdG and MDA levels, in addition to raised levels of CAT, GSH-Px, and SOD, in contrast to the Model group (*p* < 0.01). However, the level of MDA was decreased and SOD was increased in HRT group (*p* < 0.05, *p* < 0.01). MDA, CAT, and GSH-PX levels between BCD and HRT groups were statistically significant (*p* < 0.05, *p* < 0.01, *p* < 0.01) (Figures 5A–E).

### Bushen-Culuan Decoction Impacts on Ovarian mRNA Expression of Nrf2 Pathway of Premature Ovarian Insufficiency Mice Models

**FIGURE 6:**
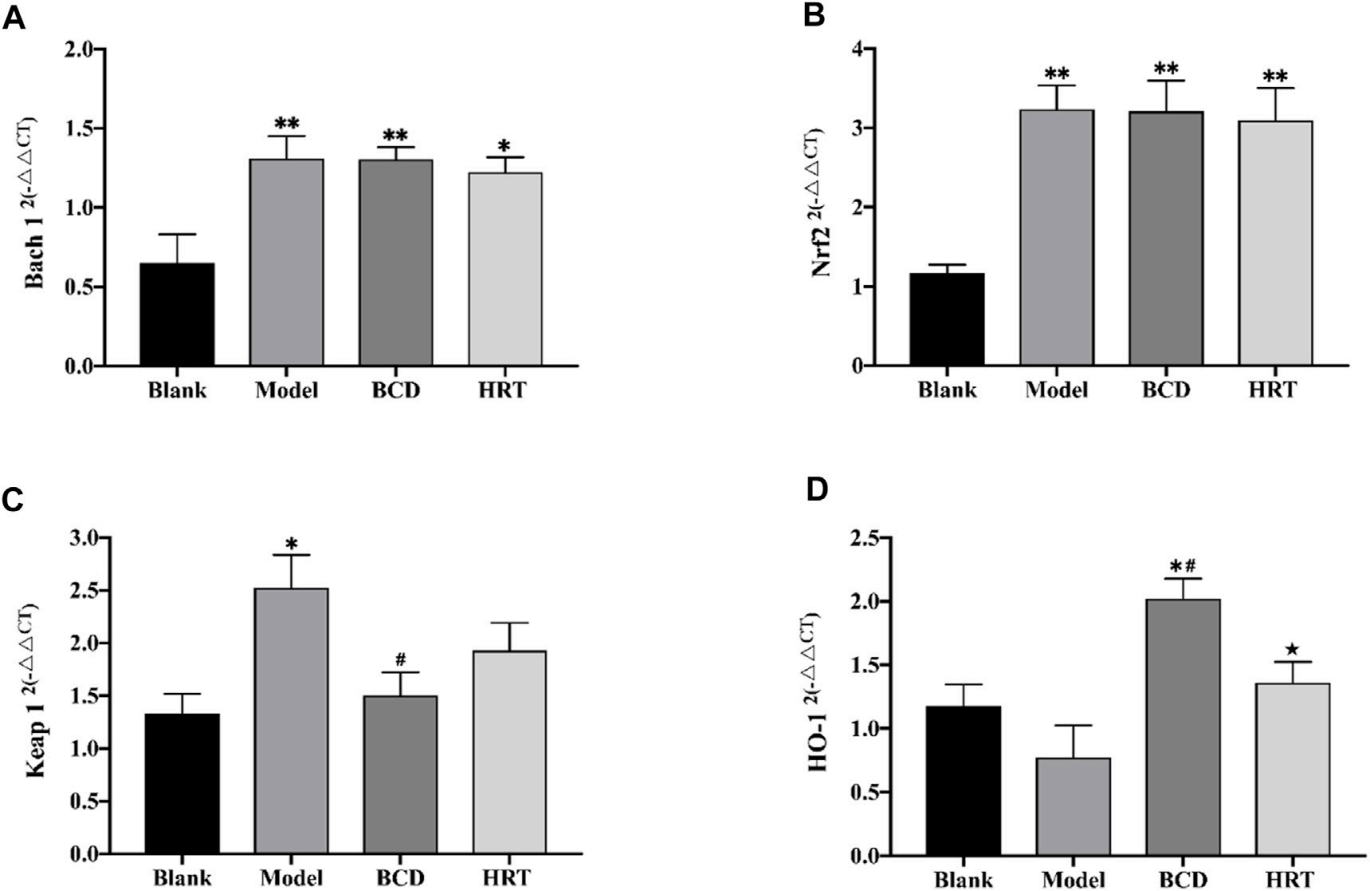
TWP activates transcription of Nrf2/Keap 1 pathway in ovaries of POI mice. The mRNA levels of Bach 1 **(A)**, Nrf2 **(B)**, Keap 1 **(C)**, and HO-1 **(D)** as assessed using by RT-PCR. The data is visualized in terms of means ± SEM. (*p < 0.05, **p < 0.01, compared with the Blank group; ^#^ *p* < 0.05, ^##^ *p* < 0.01, compared with the Model group; ★*p* < 0.05, ★★*p* < 0.01, compared with the BCD group).

Nrf2/ARE is a key signaling pathway is an important regulator of intracellular redox homeostasis. The current experiments demonstrate that TWP can induce oxidative damage, leading to depleted ovarian reserves. We utilized RT-PCR to assess various molecules involved in the Nrf2/ARE pathway. The results showed low levels of Bach 1 and Nrf2 mRNA expressions in mice of the Blank group. After TWP intervention, the mRNA expressions of Bach 1 and Nrf2 were raised in the Model, BCD and HRT groups in contrast to the Blank group (*p* < 0.01), although the differences between these three groups were not statistically significant (Figures 6A,B). Conversely, TWP exposure caused Keap 1 mRNA expression to be elevated (*p* < 0.05) and HO-1 mRNA expressions to be decreased in the Model group compared to the Blank group. Mice exposed to BCD caused lower Keap 1 mRNA expression (*p* < 0.05) but higher HO-1 mRNA expression in contrast to the Model group (*p* < 0.05). There were significant differences in terms of HO-1 mRNA expression between the HRT and BCD groups (*p* < 0.05) (Figures 6C,D).

### Bushen-Culuan Decoction Facilitates Key Proteins Nuclear Translocation of Nrf2 Pathway in Ovary of Premature Ovarian Insufficiency Mice

**FIGURE 7:**
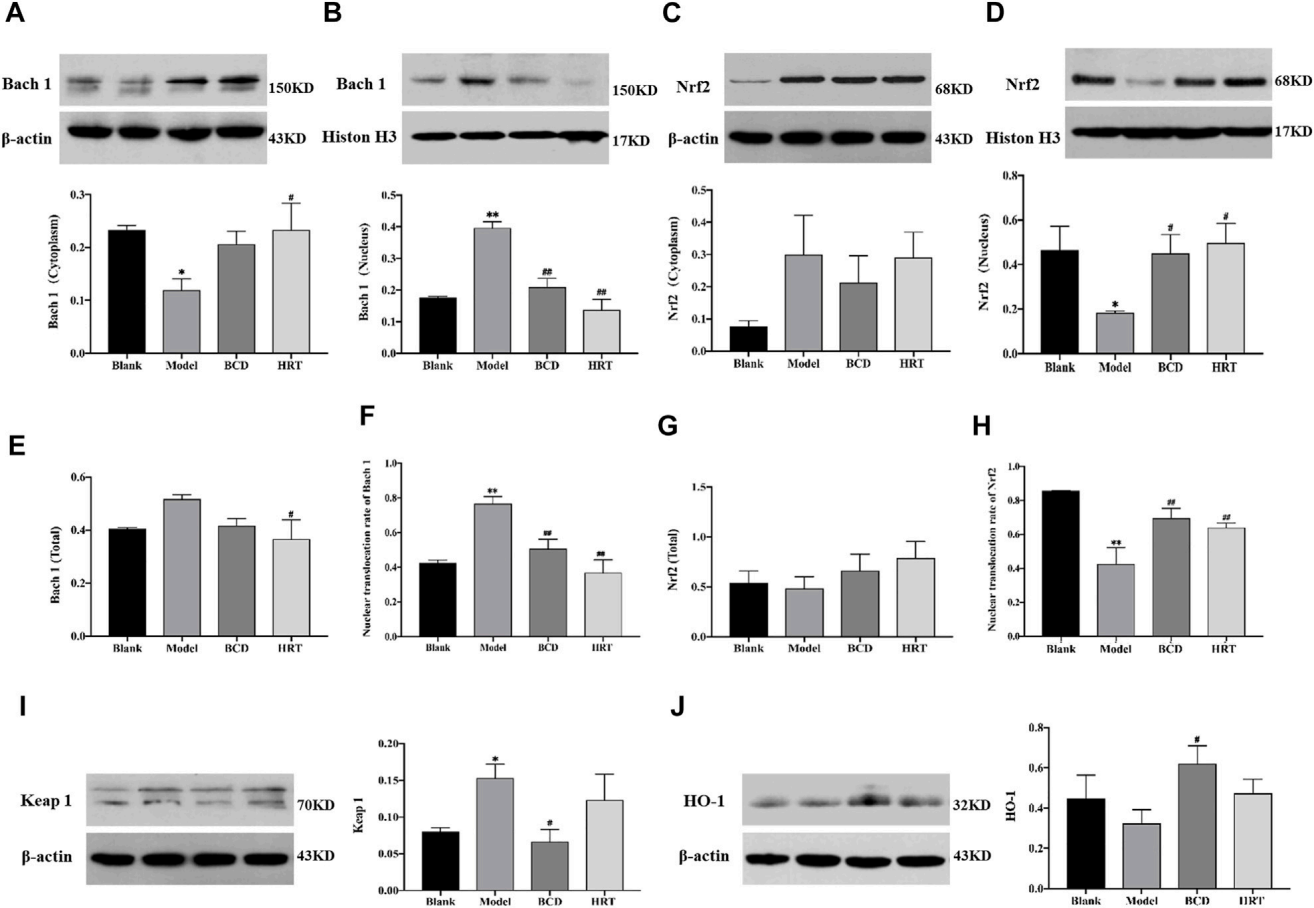
BCD facilitates nuclear translocation of Nrf2 in TWP-induced POI mice ovaries. Western blot experiments on Nrf2/Keap 1 pathway proteins were carried out. The relative protein concentrations were visualized with the ImageJ 6.0 software program. Relative quantities of Bach 1 localized in cytoplasm **(A)** and in nucleus **(B)**. The relative intensities of Nrf2 localized in cytoplasm **(C)** and in nucleus **(D)**. The total content of Bach 1 **(E)**, and the nuclear translocation rate of Bach 1 **(F)**. The total content of Nrf2 **(G)**, and the nuclear translocation rate of Nrf2 **(H)**. The relative intensities of Keap 1 **(I)** and HO-1 **(J)**. All data is a combination of eight mice from each group. The data is visualized in terms of means ± SEM. (*p < 0.05, **p < 0.01, compared with the Blank group; ^#^ *p* < 0.05, ^##^ *p* < 0.01, compared with the Model group).

Total Bach 1 and Nrf2 proteins were not significantly different between the four groups of mice. However, TWP treatment alone decreased cytoplasmic Bach 1 expression, increased nuclear Bach 1 expression (*p* < 0.05, *p* < 0.01) and increased the nuclear translocation rate of Bach 1 in the Model raised (*p* < 0.01). Compared to the Model group, Bach 1 expression in the nucleus and nuclear translocation rate were suppressed in the BCD and HRT groups (*p* < 0.01). Conversely, in the Model group, there was a rising trend of cytoplasmic Nrf2 expression but decreased nuclear Nrf2 expression (*p* < 0.05). There was also lower Nrf2 nuclear translocation rates (*p* < 0.01). After BCD and HRT treatment, Nrf2 expression in nucleus and nuclear translocation rate were raised (*p* < 0.05, *p* < 0.01) (Figures 7A–H). Furthermore, in the Model group, Keap 1 expressions were increased and decreased after BCD intervention (*p* < 0.05) (Figure 7I). HO-1 expression demonstrated a decreasing trend in the Model group, with the variability of HO-1 expressions between BCD and Model group achieving statistical significance (*p* < 0.05) (Figure 7J).

### The Animal Toxicity Test Showed That Bushen-Culuan Decoction had No Acute Reaction

**FIGURE 8:**
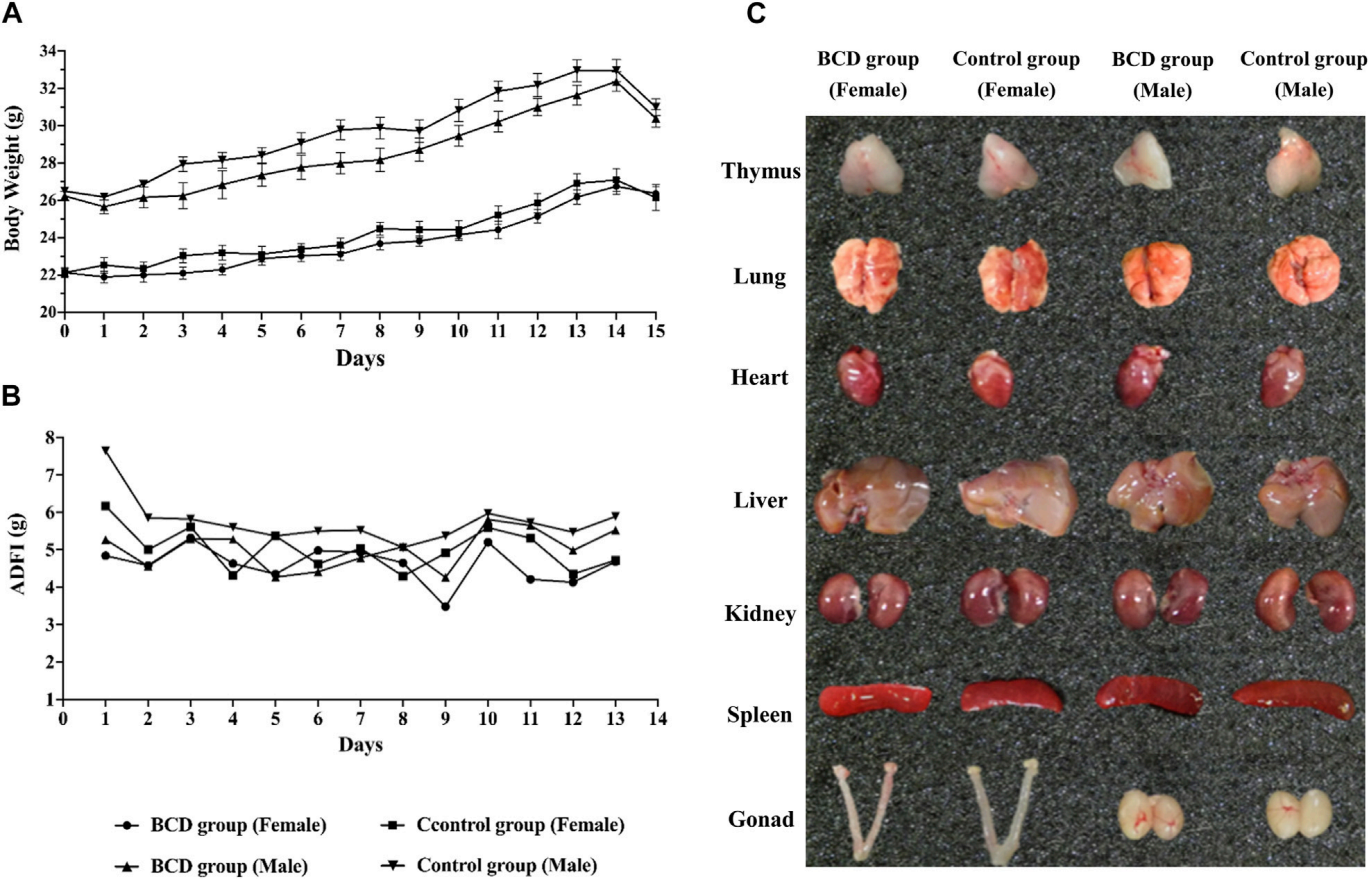
BCD has no acute toxic reaction. Acute toxicity of BCD granules investigated using the urgent toxicity testing method. **(A)** Body weight trends in BCD and control groups (including female and male mice) during the experiment. **(B)** ADFI of mice in BCD and control groups. **(C)** The gross anatomy of thymus, lung, heart, liver, kidney, spleen and gonad (female: including uterus and ovary, male: testis).

A preliminary experiment was confirmed that the dose used in the following formal toxicity tests was nonlethal. Mice of BCD group was given 5.50 g/ml BCD solution 40 ml/kg orally three times a day with an interval of 4 h between each dose. The current human clinical dosage of BCD was 2.75 g/kg. Based on the acute toxicity test, we found that the maximum oral tolerance dosage in mice was 660.38 g/kg, which was equivalent to 240 times of the human clinical dosage. During the test, none of the mice died and none developed symptoms suggestive of acute toxicity. We also demonstrated no significant difference between the two groups in terms of body weight and ADFI (Figures 8A,B). Furthermore, visual observation of internal organs revealed remarkably similar organs between the BCD and control groups (Figure 8C). Together, these results indicated that BCD exerted no significant toxicity.

## Discussion

**FIGURE 9:**
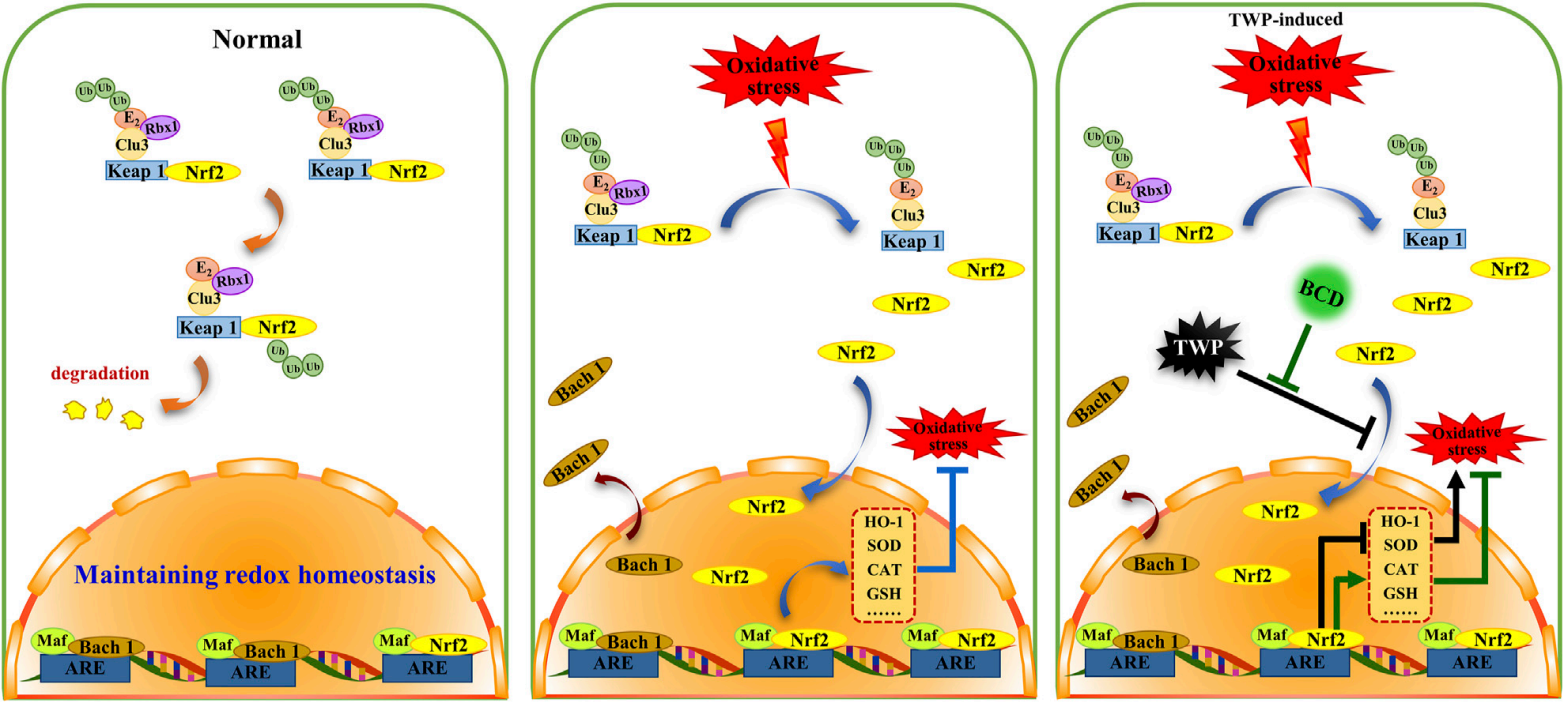
The proposed schema for BCD function in oxidative stress of TWP-induced POI. BCD prevents TWP-induced oxidative stress through regulating Nrf2/Keap 1 signaling pathway. In normal circumstances, Nrf2 is predominantly distributed in the cytoplasm, where it incorporated into a complex that binds to Keap 1 and Clu3, and then to be degradation (orange indicator). While, Bach 1 is mainly distributed in nucleus. When oxidative stress occurs, Nrf2 releases from Nrf2-Keap1-Cul3 complex, then translocates into the nucleus to combined with Maf-ARE, and subsequently promotes expressions of antioxidant enzymes, that included HO-1, SOD, CAT (blue indicator). Exposure to TWP induces ovarian oxidative stress, furthermore, TWP blocks nuclear translocation of Nrf2 and Bach 1 which ultimately inhibits the expression of antioxidant enzymes resulting in aggravation of oxidative damage (black indicator). However, with BCD co-treatment, the blocking of Nrf2 and Bach 1 nuclear translocation were reversed and the expression of antioxidant enzymes were increased (green indicator).

Despite the increasing incidence of POI, little is known regarding its exact pathogenesis. It is thought that several factors including endocrine dysfunction, immune dysregulation, and genetics may influence the occurrence of POI. Exposure to chemotherapy drugs and TWP are the two most common causes of POI in women of childbearing age. TWP is a Tripterygium wilfordii extract that exerts anti-tumour, anti-inflammatory, and immunosuppressive properties. This compound has been shown to be beneficial in autoimmune renal impairment, systemic lupus erythematosus, and rheumatoid arthritis. TWP is notorious for causing significant reproductive system toxicity, resulting in ovarian failure and dysgenesis. In addition, results from both clinical trials and animal studies indicate that TWP is an ideal drug for establishing a POI mouse model (Lu et al., 2014; Huang et al., 2018; Yuan et al., 2019). The current study uses TWP to develop a POI mouse model in order to determine the protective effects of BCD and how it protects against TWP-induced ovarian injury. Our findings show that BCD exerts protective benefits in POI mice models through antioxidant mechanisms through its actions on the Nrf2/Keap 1 signaling pathways. The proposed mechanism of BCD pharmacological actions is shown in Figure 9.

POI causes decline in ovarian function and is a state of hypergonadotropic hypogonadism. FSH, a gonadotropin, is produced in the pituitary gland and acts on FSH receptors on the granulosa cells of the ovary. FSH stimulates follicle recruitment, growth, and maturation, and is associated with E2 production. FSH modulates the female reproductive physiology and prepares the ovary for ovulation in response to the LH surge (Rimon et al., 2016; Bousfield and Harvey, 2019; Jing et al., 2019). Clinically, serum FSH and E2 levels are commonly used as measures of ovarian reserve. An abnormal increase in FSH has been associated with poor ovarian stimulation and POI. In the present study, TWP significantly increased serum FSH level, increased FSH/LH rations, and decreased serum E2 level. BCD administration notably suppressed FSH levels and FSH/LH ratios while raising E2 levels in TWP-exposed POI mice. BCD was able to reduce the degree of TWP-stimulated ovarian injury through its actions on the hypothalamus-pituitary-gonadal (HPG) axis.

AMH is a growth factor that is specifically produced by the granulosa cells of early growing ovarian follicles. AMH can regulate or suppress the number of primordial follicles to be activated and is predictive of the quantity of remnant follicles in the ovaries (Bousfield and Harvey, 2019; Pan J. et al., 2019). Since AMH is non-gonadotropin-dependent and its level remains relatively stable across the menstrual cycle, AMH can be measured at any time (Finkelstein et al., 2020; Irvin et al., 2020). Therefore, AMH is considered to be a sensitive and specific biochemical hallmark of ovarian function. Under normal circumstances, serum AMH remains at a stable level in females of childbearing age, and decreased levels are suggestive of diminished ovarian reserve (Kunt et al., 2011; Hamoda, 2017). Inhibin B is a product of ovarian granulosa cells and pituitary gland stimulation. Inhibin B is also considered a direct marker of ovarian reserve and indicates the number of developing follicles. It is a negative feedback regulator of FSH (Erdem et al., 2004; Luo et al., 2020). Our study finds that BCD treatment remarkably reverses suppressed AMH and INHB levels induced by TWP, which indicated that BCD could effectively improve ovarian function.

The normal estrous cycle comprises of four phases: proestrus, estrus, metestrus, and diestrus, all of which are governed by the controlled release of gonadal hormones such as FSH, LH, and E2 (Nallathambi and Bhargavan, 2019). The current estrous cycle stage may be derived using observed vaginal smear cell number, morphology, and types based on previous experiments. The length of the estrus cycle is primarily determined as the number of days between proestrus to the next proestrus, and generally lasts between 4 and 5 days (Cora et al., 2015; Gonzalez, 2016). The estrous cycle can be regarded as an assisted indicator of ovarian reserve. We demonstrated that TWP exerted a disruptive effect on mice estrous cycles, with BCD restoring normal cycles. Our findings are in line with those seen on gonadal hormone analyses.

It has been reported that the antral follicle count (AFC), which is the amount of ovarian antral follicles, strongly correlates with the serum AMH levels and the number of primordial follicles. The AFC serves as an important marker of ovarian reserve (Sakaguchi et al., 2019; Traversari et al., 2019). Therefore, we carried out morphometric analysis specific for distinct types of follicular populations. The use of TWP treatment alone resulted in the depletion of the primordial follicle pool. Conversely, BCD exposure improved the follicular populations across various stages. These results were in accordance with serum AMH trends in this study, further reinforcing the significant role of AMH in primordial follicle recruitment. Targeted knockout of the AMH gene caused early exhaustion of mice primordial follicle pools, leading to POI and infertility (Durlinger et al., 1999).

TWP-stimulated oxidative stress is essential in POI pathogenesis. There is a fine balance between antioxidant scavenger enzymes and reactive oxygen species (ROS) under normal circumstances. Any factor that causes oxidant accumulation or depletion of antioxidants will result in oxidative stress (Xu et al., 2018; Akino et al., 2019). The excessive ROS would irreversibly damage lipids, proteins, and DNA, causing cell oxidative damage and dysfunction (Masschelin et al., 2020). Previous studies have established 8-OHdG as an indicator of DNA damaged by oxidative stress while MDA is the end product of lipid peroxidation. Key antioxidant molecules are SOD, GSH-Px, and CAT, which protect the ovary from oxidative damage by scavenging ROS and lipid peroxides (Liu et al., 2018; Causer et al., 2020; Ghezzi, 2020). Therefore, evaluations of these indices are reflective of the degree of ovarian oxidative damage, and are markers of ovarian recovery following treatment. In accordance to previous literature, TWP increases 8-OHdG and MDA levels, while decreasing CAT, GSH-Px, and SOD levels, resulting in follicle oxidative injury and atresia (Ma et al., 2014; Fang et al., 2019). BCD treatment remarkably suppressed 8-OHdG and MDA levels while replenishing CAT, GSH-Px, and SOD levels in ovarian tissues. To conclude, we find that BCD exerted antioxidative properties and was able to rescue ovarian follicles from TWP-induced oxidative injury by reducing the degree of oxidative stress and restoring normal antioxidant enzyme levels in the ovary.

The Nrf2/Keap1 signaling pathway is a critical defence mechanism against oxidative stress (Akino et al., 2019). Nrf2 is mainly sequestered in the cytoplasm under regular circumstances and is maintained at low levels by Keap 1. Nrf2 is very sensitive to oxidative stress. Once activated, Nrf2 splits from Keap1 and moves into the nucleus to combine with ARE. The result is an upregulation of several antioxidant genes including HO-I, CAT, GSH-Px, and SOD (Akino et al., 2018; Xu et al., 2018). Oxidative stress has been shown to exert important roles in the process from oocyte maturation, fertilization, and embryo development (Agarwal et al., 2005; Wojsiat et al., 2017). Nrf2 signaling is closely associated with protection against a variety of diseases including POI, polycystic ovarian syndrome, and endometriosis, all of which strongly feature excessive oxidative stress. To further explore the likely antioxidant defense mechanisms of BCD in POI, Bach 1, Nrf2, and HO-I expressions were scrutinized in ovarian tissues.

We demonstrated that TWP treatment remarkably increased mRNA expression of Bach 1 and Nrf2, findings that mirror those of previous studies that demonstrated Triptolide, a major active ingredient extracted from TWP, to be able to induce Nrf2 overexpression in protecting against kidney cell death (Li et al., 2012). When tissues or cells are exposed to a toxic substance, there is an activation of the cellular self-adaptive defense mechanisms (Niringiyumukiza et al., 2019). Similarly, in our study, the ovary responded to TWP-induced stress by moderately increasing Nrf2 and Bach 1 mRNA levels in contrast to those of the blank group, as a self-defense mechanism. However, this self-defense mechanism was not able to completely prevent the oxidative damage caused by TWP. We found that the total protein expression of Nrf2 and Bach 1 did not significantly increase after TWP treatment. Moreover, the expression of Nrf2 in the nucleus and Bach 1 in the cytoplasm were decreased upon TWP exposure. The higher Nrf2 mRNA and relatively low Nrf2 protein expressions that we observed in the group treated with TWP alone can be explained by ubiquitination and proteasomal degradation through binding with Keap 1. These results reflected those of prior experiments, which suggested that doxorubicin induced oxidative damage by augmenting the mRNA expression levels of Nrf2 and remarkably decreasing the Nrf2 protein expression – all of which led to depleted mouse ovarian reserves (Niringiyumukiza et al., 2019). TWP appears to induce ovarian oxidative damage primarily through suppressing the nuclear translocation rates of Nrf2 protein and increasing Keap 1 expression. Treatment with BCD significantly reversed the TWP-induced low nuclear translocation rate of Nrf2, high nuclear translocation rates of Bach 1 and raised Keap 1, suggesting that the beneficial effects of BCD on TWP-stimulated ovarian reserve exhaustion may at least be partially attributed to activation of the Nrf2/Keap 1 signaling pathway, which augmented the antioxidant defense system.

## Conclusion

To conclude, the present study highlights the protective effects of BCD on TWP-triggered ovarian injury in mice. BCD may offer this protection by restoring the ovarian index, as well as AMH, INHB, and E2 levels, attenuating FSH level and FSH/LH ratio increases, improving the estrous cycle, and lastly by rescuing the primordial follicle pool through normalization of 8-OHdG, MDA, CAT, GSH-PX, and SOD levels. We hypothesize that BCD enhances oxidant defense systems and reduces oxidative damage by modifying the Nrf2/Keap 1 signaling pathway. BCD appears to be a useful therapeutic candidate in preventing and treating TWP-induced POI.

## Data Availability

The raw data supporting the conclusion of this article will be made available by the authors, without undue reservation.

## Abbreviations

AMH, anti-mullerian hormone; ARE, antioxidant response element; Bach 1, BTB and CNC homology 1; BCD, Bushen-Culuan Decoction; CAT, catalase; Cul3, Cullin3; E2, estradiol; ELISA, enzyme-linked immunosorbent assay; FSH, follicle-stimulating hormone; GSH-Px, glutathione peroxidase; HE, hematoxylin and eosin; HO-1, Heme oxygenase-1; HRT, hormone replacement therapy; INHB, inhibin-B; Keap 1, Kelch-like ECH-associated protein 1; LH, luteinizing hormone; Maf, musculoaponeurotic fibrosarcoma oncogene homolog; MDA, malondialdehyde; Nrf2, nuclear factor-erythroid 2-related factor 2; 8-OHdG, 8-hydroxy-deoxyguanosine; POF, premature ovarian failure; POI, Premature ovarian insufficiency; SOD, superoxide dismutase; TCM, traditional Chinese medicine; TWP, Tripterygium wilfordii polyglycosidium.
